# Central nervous system tumors in children under 5 years of age: a report on treatment burden, survival and long-term outcomes

**DOI:** 10.1007/s11060-022-03963-3

**Published:** 2022-02-11

**Authors:** Sarah Metzger, Annette Weiser, Nicolas U. Gerber, Maria Otth, Katrin Scheinemann, Niklaus Krayenbühl, Michael A. Grotzer, Ana S. Guerreiro Stucklin

**Affiliations:** 1 Division of Oncology and Children’s Research Center, University Children’s Hospital of Zurich, Zurich, Switzerland; 2 Division of Oncology-Hematology, Department of Pediatrics, Kantonsspital Aarau, Aarau, Switzerland; 3 Department of Pediatrics, McMaster Children’s Hospital and McMaster University, Hamilton, Canada; 4 University of Lucerne, Lucerne, Switzerland; 5 Division of Pediatric Neurosurgery, University Children’s Hospital of Zurich, Zurich, Switzerland

**Keywords:** Pediatrics, Brain tumors, Long-term outcome, Survival, Educational/occupational outcomes

## Abstract

**Purpose:**

The challenges of treating central nervous system (CNS) tumors in young children are many. These include age-specific tumor characteristics, limited treatment options, and susceptibility of the developing CNS to cytotoxic therapy. The aim of this study was to analyze the long-term survival, health-related, and educational/occupational outcomes of this vulnerable patient population.

**Methods:**

Retrospective study of 128 children diagnosed with a CNS tumor under 5 years of age at a single center in Switzerland between 1990 and 2019.

**Results:**

Median age at diagnosis was 1.81 years [IQR, 0.98–3.17]. Median follow-up time of surviving patients was 8.39 years [range, 0.74–23.65]. The main tumor subtypes were pediatric low-grade glioma (36%), pediatric high-grade glioma (11%), ependymoma (16%), medulloblastoma (11%), other embryonal tumors (7%), germ cell tumors (3%), choroid plexus tumors (6%), and others (9%). The 5-year overall survival (OS) was 78.8% (95% CI, 71.8–86.4%) for the whole cohort. Eighty-seven percent of survivors > 5 years had any tumor- or treatment-related sequelae with 61% neurological complications, 30% endocrine sequelae, 17% hearing impairment, and 56% visual impairment at last follow-up. Most patients (72%) attended regular school or worked in a skilled job at last follow-up.

**Conclusion:**

Young children diagnosed with a CNS tumor experience a range of complications after treatment, many of which are long-lasting and potentially debilitating. Our findings highlight the vulnerabilities of this population, the need for long-term support and strategies for rehabilitation, specifically tailored for young children.

**Supplementary Information:**

The online version contains supplementary material available at 10.1007/s11060-022-03963-3.

## Introduction

CNS tumors are the most common pediatric solid cancers. The average annual incidence rate is 6.18 per 100,000 in 0–4-year-olds in the U.S. [1]. This is higher than what is reported in age groups 5–9 years (5.49 per 100,000) and 10–14 years (5.83 per 100,000) [1]. In recent years, molecular profiling studies led to major advances in the understanding and classification of pediatric CNS tumors [2–4]. Development of new therapies is ongoing and expected to increase further patient survival, mitigate long-term treatment toxicities, and improve the quality of life of survivors. Notwithstanding, CNS tumors remain the most common cause of cancer-related death in children and adolescents.

Young children (< 5 years of age) are particularly prone to long-term sequelae of cytotoxic therapies [5]. Radiotherapy (RT) is associated with neurocognitive and psychological impairment, increased risk of stroke, secondary malignancies, hearing loss, and neuroendocrine deficiencies [6]. Due to its impact on the developing CNS, RT is often entirely omitted or delayed in the management of brain tumors in young children, risking suboptimal disease control. Radiation-sparing regimens used as an alternative in this population often comprise high-dose chemotherapy with stem cell rescue as a consolidation therapy or intensified intrathecal chemotherapy. Increasing the intensity of chemotherapy has led to improved disease control, however several studies report serious events including toxic deaths, mostly due to myelosuppression, sepsis and/or organ dysfunction [7–11].

Health care professionals often face the dilemma between augmenting treatment intensity for optimal disease control and minimizing acute toxicity as well as the risk of long-term sequelae [12]. Clinical outcome and quality of life of CNS tumor survivors pose a serious concern to treating physicians as well as parents, but literature focusing on young children is scarce. Here, we describe the long-term survival, health, and academic outcomes of young children at the time of CNS tumor diagnosis and treated at our institution over the last 3 decades.

## Material and methods

### Patient population

In this retrospective study, young children aged 0–5 years with a newly diagnosed primary CNS tumor and treated at the University Children’s Hospital of Zurich between January 1990 and December 2019 were identified. Date of diagnosis was defined as either date of histological confirmation of tissue sample or, if not available, date of diagnostic imaging.

The design of the study was approved by the Ethics Committee of the Canton of Zurich. A general research consent was implemented at our institution in 2015. The need for informed consent was waived for deceased patients, patients diagnosed prior to 2015 and lost to follow-up. Patients with documented refusal to participate in research were excluded.

### Clinical characteristics and long-term outcome measures

The baseline clinical characteristics included age at diagnosis, sex, tumor characteristics (histology, location, dissemination status), hydrocephalus at diagnosis, underlying genetic predisposition and treatment details. Extent of resection was determined based on neurosurgical reports and postsurgical MRI when available; if a residual tumor was described in the neurosurgical report and/or postsurgical MRI, the tumor was considered partially resected. Progression-free survival (PFS) was calculated from date of diagnosis to disease progression leading to change in treatment or death in patients without such a progression, and overall survival (OS) from date of diagnosis to death. In the absence of progression and for patients alive at last follow-up, PFS and OS were censored at the last documented date that the patient was seen by a physician (last follow-up).

The long-term health-related outcome information was extracted from the medical charts of all patients and included neurologic status, endocrine function, hearing loss, visual acuity, secondary malignancies, and cerebral vasculopathy. Ototoxicity was graded according to Chang after review of available audiograms [13]. Neurologic status was assessed during exams at regular physician’s visits and changes present in the most recent neurological examination were summarized. Neurologic deficits collected included cranial nerve deficit, motor and sensory deficits, coordination, gait, reflexes, and tonus.

At our pediatric institution, patients are followed up until 20 years of age and information on schooling and employment is regularly documented at long-term follow-up clinic visits. Academic achievement was categorized into two groups. The first group contains patients who attend regular school or work in a skilled job and the second group includes patients who attend an assisted or modified school program e.g. with smaller student numbers per class and additional assistance or who work in an unskilled or assisted job. A skilled job was defined as student with graduation after vocational training with a Federal Diploma (Fig. S3). An unskilled job includes only training on-site without graduation. Preschool-aged children were excluded from this analysis.

### Statistical analysis

Descriptive analyses were used to summarize the study population. Kaplan–Meier survival curves were generated to estimate OS probability and progression-free survival probability. Log-rank test was performed for comparison between different subgroups.

To quantify the association between different treatment modalities and long-term outcomes fisher’s exact test was used.

R version 4.0.3 and RStudio (v1.3) were used for statistical analysis. The following additional packages were used: Beeswarm, ggplot2, ggpubr, openxlsx, plotly, plotrix, plyr, survival, survminer, tableone, tidyverse, viridis.

## Results

### Patient cohort characteristics

We identified 164 children under 5 years of age and diagnosed with a primary CNS tumor between 1990 and 2019 at the University Children’s Hospital of Zurich, the largest pediatric oncology center in Switzerland. Thirty-six patients were excluded: 28 patients due to lack of sufficient information and 8 patients due to refusal to participate in research. Thus, 128 patients were included in the final analysis (Table S1).

**Fig. 1:**
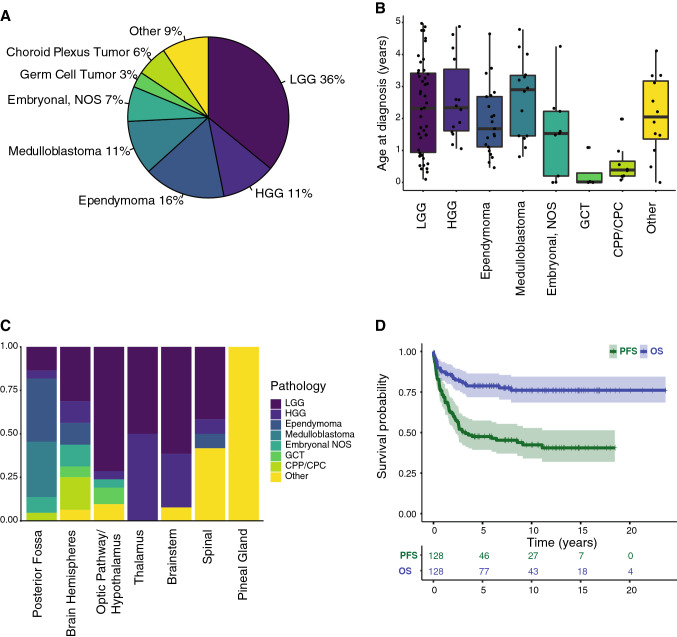
Characteristics of Study Population. **A** Tumor subgroups in percentage of whole cohort. **B** Age distribution according to tumor type. **C** Location of tumors according to tumor type. **D** Progression free survival and overall survival of whole cohort with 95%-confidence intervals and corresponding risk table. Censored cases are depicted as crosses

The main tumor subtypes were pediatric low-grade glioma (pLGG) (n = 46, 35.9%), pediatric high-grade glioma (pHGG) (n = 14, 10.9%) including five diffuse intrinsic pontine gliomas (DIPG), ependymoma (n = 21, 16.4%), medulloblastoma (n = 14, 10.9%), other embryonal tumors (n = 9, 7.0%), germ cell tumors (n = 4, 3.1%), choroid plexus tumors (n = 8, 6.2%), and others (n = 12, 9.4%) (Fig. 1A). Fourteen tumors were diagnosed based on imaging only (5 DIPG, 7 pLGG—mainly optic pathway glioma—and 2 other).

Median age at diagnosis was 1.81 years [IQR, 0.98–3.17]. Germ cell tumors (n = 4, all mature teratomas) and choroid plexus tumors (n = 8) were diagnosed in children of younger age with the eldest child being 1.09 years and 1.98 years old, respectively. The other tumor subtypes showed a more balanced distribution from 0 to 5 years of age (Fig. 1B).

The most common primary tumor site was the posterior fossa (34%), followed by the cerebral hemispheres (26%). Most tumors found in the optic pathway and hypothalamus/midline region were pediatric low-grade gliomas (n = 15, 71%), 3 of those patients were diagnosed with neurofibromatosis 1 (NF1). Tumors located in the thalamus and brainstem were mostly pediatric low-grade (n = 9, 56%) or high-grade gliomas (n = 6, 38%) (Fig. 1C). Spinal tumors (n = 12) included 5 pLGG, 1 pHGG, 1 ependymoma, and 5 others (Ewing sarcoma, undifferentiated sarcoma, ganglioneuroma, juvenile xanthogranuloma, and plexiform neurofibroma).

### Treatment modalities

Most patients (n = 112, 87.5%) underwent surgery, in 41% gross total resection (GTR) was achieved (Table S1). Fifty percent of patients had an uneventful postoperative course, for 9% there was no detailed information available. Seven patients (6.3%) had an ischemic event perioperatively, 5 (4.5%) presented with hemorrhages in the perisurgical period, with one being hemodynamically relevant. Ten patients (8.9%) developed hygroma or craniospinal fluid (CSF) leakage, 11 (9.8%) had nerve lesions with subsequent (transient) paresis in the corresponding area and 18 (16%) had other postoperative complications such as epilepsy, hemisyndromes, and endocrine deficiencies. Some patients suffered multiple complications.

Sixty-three percent of patients received chemotherapy, most of them enrolled on or treated as per tumor-specific protocols (Fig. S2; Table S2) [14, 15].

Radiotherapy was performed in 39.8% of patients (n = 51), either upfront (n = 27) or at progression (n = 24), two of those 24 were re-irradiated after further progression. The majority (56.9%) underwent proton radiation (introduced in Switzerland in 1996), 37.3% photon radiation, and 5.9% both modalities. Almost 30% underwent craniospinal radiation.

### Survival outcome

Median follow-up time of surviving patients was 8.39 years [range 0.74–23.65 years]. Median progression-free survival (PFS) of the whole cohort was 3.3 years [95% CI, 2.3–NA]. The 5- and 10-year OS probability was 78.8% resp. 76.1% for the whole cohort (Fig. 1D).

**Fig. 2:**
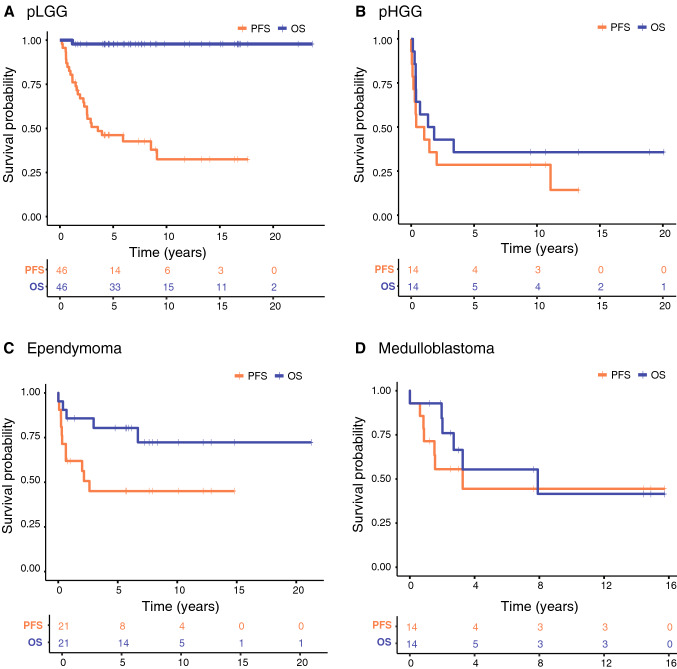
Kaplan–Meier Curves **A**–**D** depicting progression free survival and overall survival of the four main tumor subgroups with corresponding risk-tables. Censored cases are depicted as crosses

The estimated 10-year OS for patients with pLGG was 97.8% (95% CI, 93.7–100) (Fig. 2A) with a PFS after 5 and 10 years of 46.2% (95% CI, 33.6–63.5) and 32.5% (95% CI, 19.3–54.7). Patients with pHGG had a 5- and 10-year OS probability of 35.7% (95% CI, 17.7–72.1) (Fig. 2B), with a 5- and 10-year PFS of 28.6 (95% CI, 12.5–65.4). Five-year OS was 80.4% (95% CI, 64.8–99.7) and 10-year OS was 72.3% (95% CI, 53.7–97.5) in patients with ependymoma (Fig. 2C) with a PFS of 45% (95% CI, 27.5–73.7) after 5 and 10 years. Medulloblastoma patients had a 5-year OS of 55.4% (95% CI, 32.2–95.4), and a 10-year OS of 41.5% (95% CI, 19–91) (Fig. 2D). The 5- and 10- year PFS probability of patients with medulloblastoma was 44.4% (95% CI, 23.2–85.2).

Most of the deceased patients died within the first 5 years after diagnosis. Of those patients who died, the majority (25 of 28, 92.8%) died of tumor progression/relapse. One patient initially diagnosed with medulloblastoma died after diagnosis of radiation-induced high-grade glioma. The cause of death was undocumented or unclear for two patients.

### Long-term health-related outcomes

#### Neuroendocrine sequelae

Twenty-six patients (20.3%) presented with endocrine deficiencies at last follow-up, where 21 (81%) received hormonal replacement therapy. Most (85%) presented with hypopituitarism. Hypopituitarism was associated with radiotherapy [odds ratio (OR) 4.51, 95% CI 1.58–14.24] and chemotherapy (OR 3.27, 95% CI 0.99–14.13) (Table S3). Other endocrine alterations included obesity or precocious puberty.

Fourteen patients were over 18 years of age at last follow-up. As far as documented none of them conceived a child. Four patients (4 of 8 females) had ovarian insufficiency and two required estradiol replacement. For the male patients (n = 6), spermiograms were not available.

#### Visual outcomes

Visual impairment is multifactorial and prevalent also in the general population. Focusing on the subset of patients with optic pathway glioma and tumor-associated visual impairment, all patients (n = 13; 2/13 patients with NF1) with optic pathway glioma showed some degree of visual impairment at last follow-up and four patients were blind in at least one eye.

#### Hearing outcome

Sixteen patients (12.5%) had hearing impairment at last follow-up (≧ Chang Grade 2b) (Table 1). We found an association between hearing impairment and platinum containing chemotherapy (OR 6.898, 95% CI 1.457–65.911) and radiotherapy (OR 3.915, 95% CI 1.155–15.435) (Table S3).

**Table 1:** Health-related outcomes for each tumor subgroup

	LGG	HGG	Ependymoma	Medulloblastoma	Embryonal tumor	Germ cell tumor	Choroid plexus tumor	Other	Total n (%)
n	46	14	21	14	9	4	8	12	128 (100)
Age at diagnosis years (median [IQR])	2.32 [0.94, 3.41]	2.34 [1.62, 3.54]	1.68 [1.11, 2.68]	2.90 [1.46, 3.35]	1.53 [0.20, 2.22]	0.02 [0.00, 0.30]	0.39 [0.20, 0.66]	2.05 [1.36, 3.17]	
Any abnormal neurological finding (%)	26 (56.5)	10 (71.4)	12 (57.1)	10 (71.4)	6 (66.7)	2 (50.0)	6 (75.0)	7 (58.3)	79 (61.7)
Seizures (%)	10 (21.7)	3 (21.4)	1 (4.8)	4 (28.6)	5 (55.6)	1 (25.0)	2 (25.0)	2 (16.7)	28 (21.9)
Neuroendocrine sequelae (%)	10 (21.7)	1 (7.1)	6 (28.6)	4 (28.6)	2 (22.2)	1 (25.0)	1 (12.5)	3 (25.0)	28 (21.9)*
Hearing outcome (%)									
Chang Grade 0	39 (84.8)	12 (85.7)	13 (61.9)	10 (71.4)	8 (88.9)	3 (75.0)	7 (87.5)	12 (100.0)	104 (81.2)
Chang Grade 1b	1 (2.2)	0 (0.0)	2 (9.5)	2 (14.3)	0 (0.0)	0 (0.0)	0 (0.0)	0 (0.0)	5 (3.9)
Chang Grade 2a	2 (4.4)	0 (0.0)	0 (0.0)	0 (0.0)	0 (0.0)	0 (0.0)	0 (0.0)	0 (0.0)	2 (1.5)
Chang Grade 2b	1 (2.2)	0 (0.0)	0 (0.0)	0 (0.0)	0 (0.0)	1 (25.0)	1 (12.5)	0 (0.0)	3 (2.3)
Chang Grade 3	1 (2.2)	1 (7.1)	1 (4.8)	2 (14.3)	0 (0.0)	0 (0.0)	0 (0.0)	0 (0.0)	5 (3.9)
Chang Grade 4	1 (2.2)	1 (7.1)	5 (23.8)	0 (0.0)	1 (11.1)	0 (0.0)	0 (0.0)	0 (0.0)	8 (6.2)

*Including 2 patients with transient endocrine alterations

#### Secondary malignancies

Three patients were diagnosed with a secondary malignancy during follow-up (Table 2). All of them had been irradiated by either proton or photon radiation, received chemotherapy, and underwent surgery. None of them had a known underlying genetic predisposition, including NF1. Importantly, the patient with ATRT and later MPNST had no evidence of germline alterations in *SMARCB1*, *SMARCA4*, *NF1* or *TP53*. One patient succumbed to his secondary malignancy.

**Table 2:** Characteristics of patients with diagnosis of a secondary malignancy

Age at diagnosis of first tumor (years)	Sex	Pathology primary tumor	Deceased	OS (years)	Surgery	Chemo	Radiation	Time from first diagnosis to second (years)	Type of secondary malignancy
2.3	Male	Fibrillary astrocytoma	No	16.9	Yes, GTR	Yes	Yes, Photon	9.2	T-ALL
1.5	Female	ATRT	No	12.2	Yes, STR	Yes	Yes, Proton	10.5	Malignant peripheral nerve sheath tumour (outside of radiation field)
3.4	Male	Classic medulloblastoma	Yes	3.3	Yes, GTR	Yes	Yes, Photon	3.2	High grade diffuse brainstem glioma

#### Cerebrovascular disease

Two out of 51 irradiated patients (3.9%) presented with a moyamoya vasculopathy during follow-up. Both had been diagnosed with posterior fossa ependymoma and had received focal proton radiation at below 4 years of age, with a dose to the tumor bed of 54 Gy and 59.4 Gy, respectively.

Nine patients (7%) experienced a stroke during follow-up time, in four patients after surgical resection of the tumor and in 2 patients stroke was deemed related to radiation-induced cerebrovascular disease (2.5 years and 13 years post completion of radiation).

In summary, among survivors followed for more than 5 years (n = 77), 87% present with any tumor- or treatment-related sequelae, 61% had any neurological deficit, 30% presented with endocrine sequelae and 81% of them with need for hormone replacement, 17% with hearing impairment, and 56% with visual impairment at last follow-up.

### Education and occupational outcomes

**Fig. 3:**
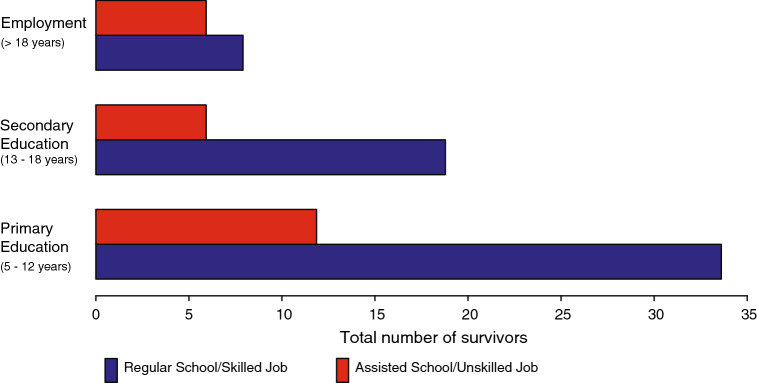
Educational/occupational outcomes by age at last follow-up

Thirty-seven patients had not yet reached school age at time of last follow-up. Information on schooling was not available for 6 patients. Of the remaining 85 patients, 71.8% were able to attend regular school and/or work in a skilled job, whereas 28.2% were schooled in a modified program or working in an unskilled or assisted job (Fig. 3). Of the patients over 18 years of age at last follow-up (n = 14), one patient attended university. Fifteen of 68 (22%) patients 1 year or older at diagnosis were schooled in a special school environment or working in an unskilled job, compared to 9 out of 17 (53%) patients under 1 year of age at diagnosis. Despite limited patient numbers, this trend suggests lower academic achievement in children diagnosed in the first year of life.

## Discussion

Among survivors of childhood cancer, the cumulative burden of chronic health conditions is highest in patients diagnosed with CNS malignancies [16]. Providing treatments which are both efficient and of acceptable toxicity is thus a great challenge in pediatric neuro-oncology, especially when treating young children. A report from the Childhood Cancer Survivor Study (CCSS) suggested that treatment transformation over the past decades has already decreased the overall treatment burden [17]. Nevertheless, the long-term impact of therapy in the immature CNS and in the developing child remains a major concern. In addition, CNS tumors in young children often display distinct clinical and biological features when compared to those in older children and adolescents [18–21]. In this study we report the survival, health-related and educational outcomes of young children with CNS tumors treated at a tertiary pediatric oncology center over a period of three decades. Importantly, our study provides long follow-up and comprehensive clinical outcome data on a large cohort of sequentially diagnosed, unselected patients in this age group. We found that mortality was largely due to tumor progression/relapse and predominantly within the first 5 years after diagnosis. The high prevalence of tumor- and treatment-related sequelae (87%) highlights the need for close monitoring and long-term, multidisciplinary support strategies.

With a 5-year OS of 78.8%, the survival outcome of our cohort is comparable to previous studies, albeit differences regarding patient characteristics and therapy between studies [1, 22–24]. One study including 35 children under 1 year of age at diagnosis reported a considerably lower 5-year OS of 57% [25]. The OS of pLGG in our cohort seems to be better than reported in previous studies [20, 21], which were multi-institutional and thus more heterogenous in treatment approach. PFS was defined in our study as time from diagnosis to a progression leading to change in treatment. This may overestimate PFS compared to other studies with other definitions and should be considered when interpreting our results, especially for patients with pLGG.

Other studies reported a higher mortality rate in children diagnosed under 1 year of age when compared to older children [20, 26, 27]. Despite a trend in literature towards worse outcome in children diagnosed in the first year of life, we could not confirm this finding in our cohort, noting however a trend towards worse OS in children diagnosed in the first 6 months of life (Fig. S1) [28]. This may be explained by differences in patient characteristics (age at diagnosis limited to 5 years or younger in this study) and tumor distribution (e.g. lower numbers of patients with ATRT in our cohort).

Survival rates of children diagnosed with a CNS tumor seem to have increased over the last years [23, 24], while the prognosis for high grade subtypes remains poor. Similarly, despite encouraging survival rates of the whole cohort, patients with pHGG and medulloblastoma had an unfavorable outcome, with a 5-year OS of 35.7% and 55.4%, respectively.

A study summarizing the outcomes of a cohort of 20 survivors, previously diagnosed with a brain tumor in the first year of life, reported 70% with neurological dysfunction, 25% with endocrine dysfunction, 15% with hearing impairment, and 45% with visual impairment [25]. These numbers are comparable to our findings, despite differences in age of inclusion. A report from the CCSS showed that patients diagnosed with a CNS malignancy had a significantly higher risk of developing a chronic health condition 5 years after diagnosis when compared to their siblings, including endocrine, neurologic, or sensory deficits [29]. Hearing impairment was associated with radiation therapy and platinum chemotherapy, both known risk factors for sensorineural hearing loss [30, 31]. A previous study from the CCSS reported hearing impairment in 12% of childhood CNS tumors survivors [30]. A study conducted at St. Jude Children’s Hospital compared survivors of CNS tumors to survivors of non-CNS tumors exposed to high-risk ototoxic cancer therapy and reported a prevalence of 36% of severe hearing loss in CNS tumor survivors. There was no association between age at diagnosis and hearing loss [32].

Hypopituitarism was associated with radiotherapy, as previously described [33–35]. A recent study identified younger age, tumor location, and radiotherapy as relevant risk factors for developing hypothalamic-pituitary disease [36]. Due to lack of sufficient information, an analysis on radiation dose-dependent outcomes was not possible in our cohort. A report from the CCSS could not find a dose-dependent risk elevation in radiotherapy for endocrine deficits [37]. The important issue of fertility could not be addressed in a uniform manner in our cohort and should be addressed in future studies.

We found that most (71.8%) of our school-aged patients or older at the time of analysis attended regular school or were employed in a skilled job. This is higher than what a previous study reported including children diagnosed with a CNS tumor in the first year of life [25]. A recent study with children aged 4 years or younger at diagnosis and treated with proton radiotherapy described a rate of 90% of children functioning in regular schools, 46% of them followed a specialized educational plan, whereas 23% and 36% had a classroom aid or outside tutor, respectively [38]. Interestingly, recent analyses of childhood cancer survivors showed that especially CNS malignancy, younger age at diagnosis, and radiotherapy increased the risk of unemployment and lower educational attainment [39–41]. Another analysis showed that childhood survivors of CNS tumors were less likely to complete high school than their siblings but could lower that risk by using special education support [42]. These findings further highlight the need to develop toxicity-sparing treatments and provide long-term support for these patients.

The size and heterogeneity of our study population, as well as limitations associated with the retrospective nature of the study, need to be considered when interpreting our findings. Assessment of long-term outcome has not followed a standard procedure in terms of frequency and tools used, limiting interpatient comparisons. The classification of CNS tumors has undergone several important revisions over the decades covered in this study. The main tumor entities have been reclassified and subdivided into new subgroups, reflecting the heterogeneity in tumor biology, especially in pediatric brain tumors in young children [3, 43]. Though molecular profiling was beyond the scope of this study, future studies dissecting correlation between tumor biology and health-related outcome will be critical to understand the impact of tumor biology on long term functional outcomes. A further limitation of our study is lack of comparison cohort of older pediatric and adolescent patients diagnosed with CNS tumors. Nevertheless, our findings provide a comprehensive overview of an unselected large cohort of patients, diagnosed and followed by a multidisciplinary team in a tertiary center over a period spanning 3 decades.

## Conclusions

Young children are at a high risk for long-term morbidity after diagnosis of a CNS tumor. Encouragingly, though a vast proportion of survivors experience health-related sequelae, most were integrated in regular schools. Our study highlights the importance of long-term support strategies, tailored to young children. These include early screening for visual and hearing impairment, as well as endocrinopathy and neuropsychology assessments, to offer appropriate support. Advances in treatment modalities, including targeted anti-tumor therapies and improvement in high-precision radiation techniques, will hopefully lead to a further reduction in treatment burden and better long-term outcomes in these children.

## Supplementary Information

Below is the link to the electronic supplementary material.

Supplementary file1 (DOCX 607 KB)

## Data Availability

The datasets generated during and/or analysed during the current study are available from the corresponding author on reasonable request.
