# Correction to: Central nervous system tumors in children under 5 years of age: a report on treatment burden, survival and long-term outcomes

**DOI:** 10.1007/s11060-022-03976-y

**Published:** 2022-02-28

**Authors:** Sarah Metzger, Annette Weiser, Nicolas U. Gerber, Maria Otth, Katrin Scheinemann, Niklaus Krayenbühl, Michael A. Grotzer, Ana S. Guerreiro Stucklin

**Affiliations:** 1 Division of Oncology and Children’s Research Center, University Children’s Hospital of Zurich, Zurich, Switzerland; 2 Division of Oncology-Hematology, Department of Pediatrics, Kantonsspital Aarau, Aarau, Switzerland; 3 Department of Pediatrics, McMaster Children’s Hospital and McMaster University, Hamilton, Canada; 4 University of Lucerne, Lucerne, Switzerland; 5 Division of Pediatric Neurosurgery, University Children’s Hospital of Zurich, Zurich, Switzerland

## Correction to: Journal of Neuro-Oncology https://doi.org/10.1007/s11060-022-03963-3

1. The first sentence of the subsection Secondary malignancies (in Results) should read as follows:

Three patients were diagnosed with a secondary malignancy during follow-up (Table 2).

(2) In Table 1, the following corrections were required:
  - the percentage of patients with seizures specified in the last column should be 21.9 (for n = 28).
  - the total number of patients specified in the last column for Neuroendocrine sequelae should be qualified to include two patients with transient endocrine alterations.
  - the percentage of LGG patients with Grade 0 hearing deficits should be 84.8 (for n = 39).

