# Supplementary material for: Central nervous system tumors in children under 5 years of age: a report on treatment burden, survival and long-term outcomes
Source: J Neurooncol. 2022 Feb 11;157(2):307–17. doi: 10.1007/s11060-022-03963-3 (PMC9021074; doi:10.1007/s11060-022-03963-3)
Supplement: Supplementary file 1 — Supplementary file1 (DOCX 607 KB) [file 11060_2022_3963_MOESM1_ESM.docx]

**Supplemental information for Article: “Central Nervous System Tumors in Children under 5 Years of Age: A Report on Treatment Burden, Survival and Long-Term Outcomes”**

**Authors:** Sarah Metzger, Annette Weiser, Nicolas Gerber, Maria Otth, Katrin Scheinemann, Niklaus Krayenbühl, Michael A. Grotzer, Ana S. Guerreiro Stucklin

**Table S1: Patient population characteristics.**

| All Patients, N | 128 |
| --- | --- |
| Age at diagnosis, median [IQR] | 1.81 [0.98, 3.17] |
| SEX = Female (%) | 57 (44.5) |
| Hydrocephalus at diagnosis (%) | 60 (46.9) |
| VP Shunt (%) | 41 (32.0) |
| Surgery (%) | 112 (87.5) |
| Biopsy | 9 |
| Subtotal Resection | 57 |
| Gross total resection | 46 |
| Chemotherapy (%) | 81 (63.3) |
| Radiation (%) | 51 (39.8) |
| Proton | 29 |
| Photon | 19 |
| Both | 3 |
| Underlying Genetic Disease | 9 (7.0) |
| Neurofibromatosis 1 | 3 |
| PTEN Hamartoma Tumor SyndromE | 2 |
| Other | 4 |

**Fig. S1: Comparison of Overall Survival of Patients according to Age Group.**


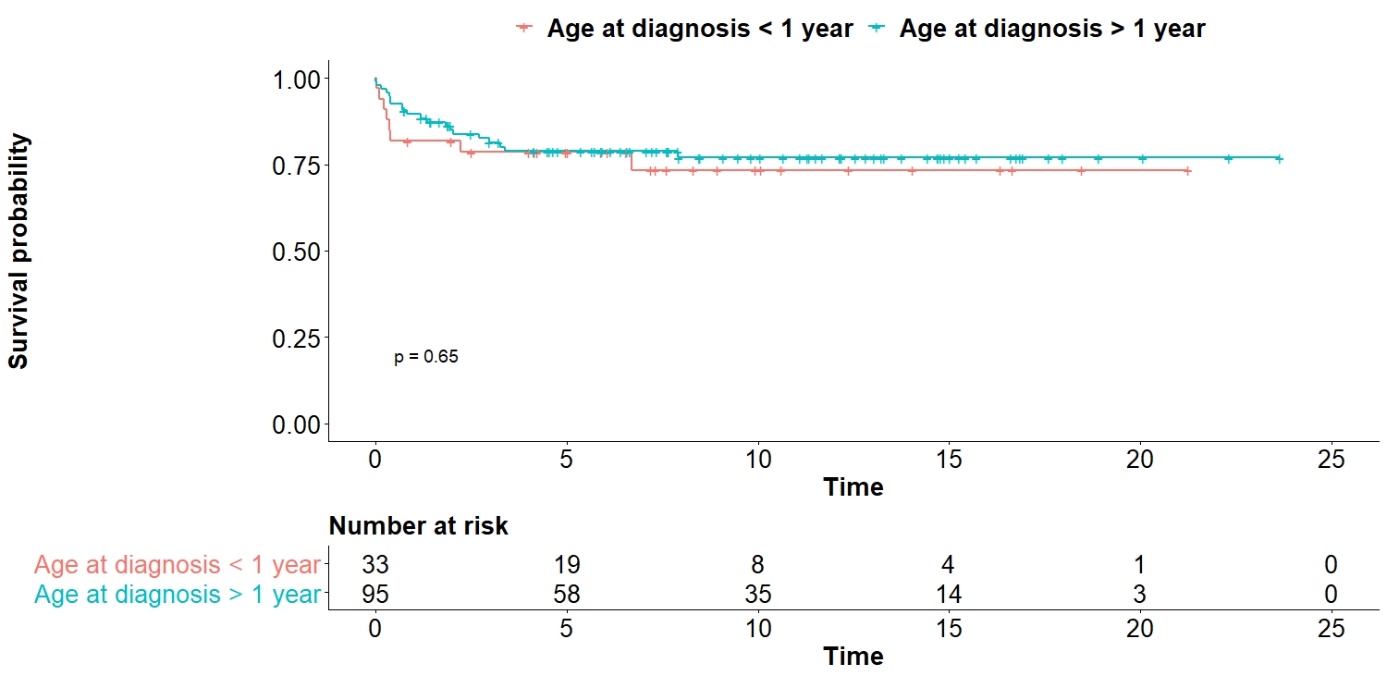

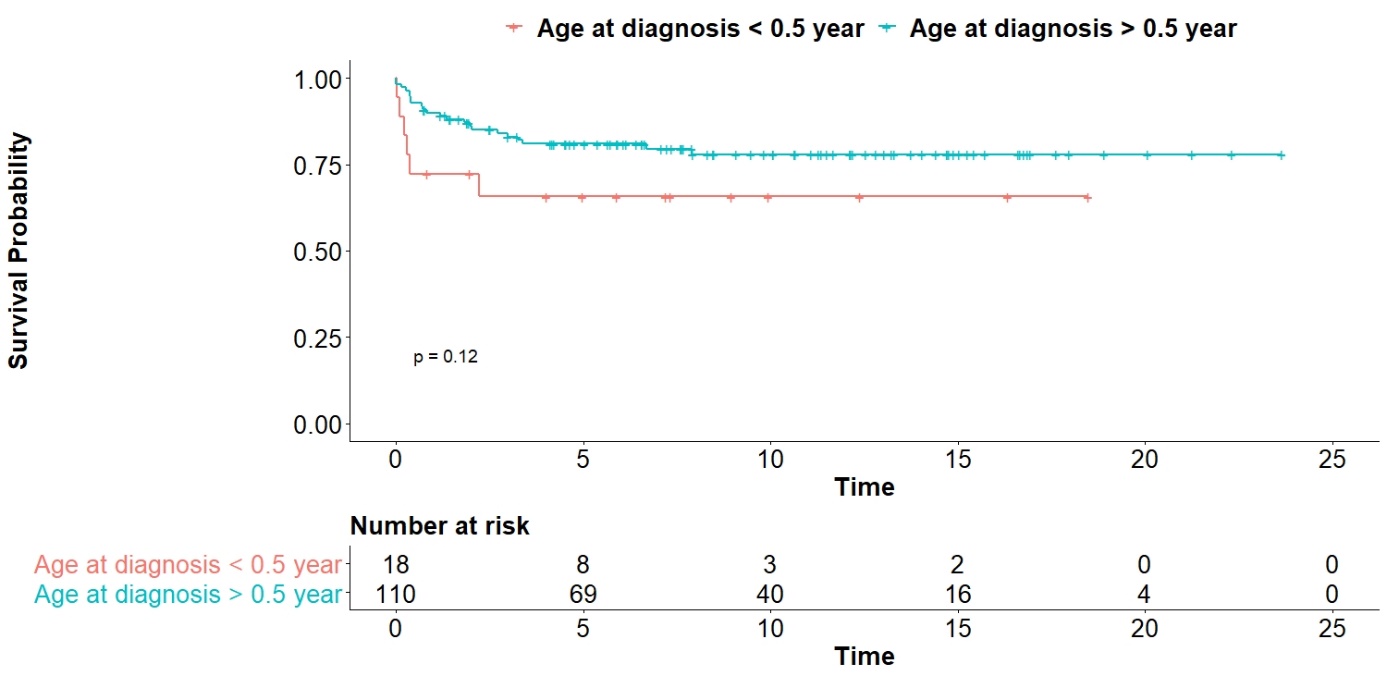


**Fig.S2: Treatment Strategies over the Decades^[[1]](#footnote-1)^**

Treatment protocols:

- HIT 2000
- LGG SIOP/GPOH 96/2004
- MAKEI 96
- EuroEwing 99
- CWS 2002
- *as per* DFC I/IRS III Boston

Treatment protocols:

- HIT 2000
- SIOP LGG 2004
- I-HIT MED registry/HIT MED Guidance
- EU-RHAB
- SIOP PNET 5 MB
- SIOP ependymoma II
- CPT 2009
- SIOP

Treatment protocols:

- POG 9233-Baby-POG 2
- LGG SIOP/GPOH 96

Patients diagnosed at Kinderspital Zurich from 1990-2019, <5 years of age, meet inclusion criteria

n = 128

1990-1999

n = 16*

2010-2019

n = 57

2000-2009

n = 55

| Date of Diagnosis | **1990-1999** | **2000-2009** | **2010-2019** |
| --- | --- | --- | --- |
| Total n | 16 | 55 | 57 |
| **Pathology**  pLGG (%)  pHGG (%)  Ependymoma (%)  Medulloblastoma (%)  Other (%) | 7 (44)  2 (12)  1 (6)  3 (19)  3 (19) | 17 (31)  5 (9)  7 (13)  3 (5)  23 (42) | 22 (39)  7 (12)  13 (23)  8 (14)  7 (12) |
| **Surgery (%)**  VP Shunt (%)  VP Shunt Revision (%) | 15 (94)  5 (31)  3 (19) | 49 (89)  18 (33)  7 (13) | 48 (84)  18 (32)  6 (11) |
| **Chemotherapy (%)**  Platinum-containing CT (%)  Intrathecal CT (%)  Highdose CT with stemcell rescue (%) | 10 (63)  7 (44)  0 (0)  0 (0) | 32 (58)  22 (40)  9 (16)  5 (9) | 39 (68)  37 (65)  7 (12)  7 (12) |
| **Radiotherapy (%)**  Proton (%)  Photon (%)  CSI (%)  Median Age at radiation (years, range) | 6 (38)  0 (0)  6 (38)  2 (12)  5.0 (3.47-7.09) | 22 (40)  12^[[2]](#footnote-2)^ (22)  11 (20)  7 (13)  3.9 (1.74-13.06) | 23 (40)  20^[[3]](#footnote-3)^ (35)  5 (9)  6 (11)  3.2 (0.84-6.81) |

**Table S2: Patient Population Divided by Decades**

**Fig. S3: Swiss Education System^[[4]](#footnote-4)^**

Terciary

level

Secondary

level

Primary

level

*Primary level / Compulsory School*: 11 years, children typically aged 5-15 years (variable according to individual curriculum/canton)

*Secondary / Higher Educational Level*: Typically, from age 16-19 years, includes:

- *Vocational education (apprenticeship)*: training on the job, accompanied by theoretical training in school, leading to a skilled job with possibility to extend to higher vocational education

- *Specialised baccalaureate*: towards admission for University of Applied Sciences, University of Teacher Education, Nursing School etc. (Bachelor/Master)

- *Baccalaureate:* towards admission for University/Federal Institutes of Technology (Bachelor, Master, PhD, Doctorate)

In Switzerland, about 2/3 of students undergo vocational education, and 1/3 attend *S*pecialised baccalaureate or Baccalaureate education.^[[5]](#footnote-5)^

**Table S3: Associations of Sequelae and Treatment Modalities with Fisher’s exact test**

| **Deficits/Sequelae** | **Radiation** | **Chemotherapy** | **Surgery** |
| --- | --- | --- | --- |
| **Hypopituitarism** | OR 4.51  95%-CI 1.58-14.24  P = 0.002 | OR 3.27  95%-CI 0.99-14.13  P = 0.05 | OR Inf  95% CI 0.90 - Inf  P = 0.07 |
| **Hearing Impairment at last follow-up** | OR 3.92  95% CI 1.15-15.44  P = 0.015 | Platinum-containing  OR 6.90  95%-CI 1.46-65.91  P = 0.005  All Chemotherapy  OR 4.65  95%-CI 0.99-44.18  P= 0.049 | OR 2.31  95%-CI 0.31-104.03  P = 0.691 |
| **Cerebrovascular Events** | OR 1.14  95%-CI 0.43-2.96  P = 0.824 | OR 1.74  95%-CI 0.63-5.37  P = 0.266 | OR 1.12  95%-CI 0.27-6.23  P = 1 |
| **Abnormal Neurological Finding at last follow-up** | OR 1.08  95%-CI 0.49-2.39  P = 1 | OR 0.87  95%-CI 0.38-1.94  P = 0.85 | OR 0.70  95%-CI 0.18-2.39  P = 0.59 |
| **Visual Impairment at last follow-up** | OR 1.46  95%-CI 0.68-3.19  P = 0.36 | OR 2.58  95%-CI 1.17-5.84  P = 0.016 | OR 0.87  95%-CI 0.26-2.82  P = 1 |

1. * A greater proportion of patients diagnosed in the earlier decade was excluded due insufficient/ missing information [↑](#footnote-ref-1)
2. 1 patient received proton and photon therapy (counted separately to each category) [↑](#footnote-ref-2)
3. 2 patients received proton an photon therapy (conted separately to each category) [↑](#footnote-ref-3)
4. <https://www.sbfi.admin.ch/sbfi/en/home/education/swiss-education-area/swiss-education-system.html> (21.01.2022;20:54, MET) [↑](#footnote-ref-4)
5. <https://www.edk.ch/de/bildungssystem/beschreibung> (21.01.2022;20:54, MET) [↑](#footnote-ref-5)
